# Efficacy and safety of ultrasound-guided microwave “Near-Total Ablation” for refractory hyperthyroidism: a retrospective study

**DOI:** 10.3389/fendo.2026.1935967

**Published:** 2026-09-29

**Authors:** Fenglin Wu, Shicong Song, Junyu Xue, Suting Liu, Jia Huang, Wenwei Xu, Lin Zhou

**Affiliations:** 1 Department of Endocrine and Metabolism, Nanfang Hospital, Southern Medical University, Guangzhou, Guangdong, China; 2 Department of Geriatrics, Nanfang Hospital, Southern Medical University, Guangzhou, Guangdong, China

**Keywords:** Graves’ disease, microwave ablation, Near-Total Ablation, refractory hyperthyroidism, ultrasound guidance

## Abstract

**Objective:**

The current study aims to investigate the efficacy and safety of ultrasound-guided microwave “Near-Total Ablation” in the treatment of refractory hyperthyroidism, with a particular focus on the postoperative drug-free euthyroidism rate and the incidence of hypothyroidism.

**Methods:**

A total of 60 patients with refractory hyperthyroidism who met the clinical diagnostic criteria for refractory hyperthyroidism were enrolled in this study. All patients underwent ultrasound-guided microwave “Near-Total Ablation” targeting the bilateral thyroid lobes and isthmus.

**Results:**

Of the 60 enrolled patients, 57 were included in the postoperative follow-up, while 3 were excluded. The postoperative follow-up duration ranged from 6 to 36 months, with a mean of 10.6 ± 4.6 months. During follow-up, 35 patients achieved hyperthyroidism remission (remission group), with a remission rate of 61.4%, while 22 patients had unrelieved hyperthyroidism (non-remission group), with a non-remission rate of 38.6%. In the remission group, 4 patients with decreased thyroid-stimulating hormone (TSH) and 8 patients with recurrent hyperthyroidism received one session of supplementary ablation. In the non-remission group, 18 patients received one session of supplementary ablation, 3 patients received two sessions, and 1 patient received three sessions. At the final follow-up, the remission rate of hyperthyroidism was 98.2% (56/57), with a non-remission rate of 1.8% (1/57). The rate of drug-free euthyroidism was 68.4% (39/57), and the incidence of hypothyroidism was 29.8% (17/57). The recurrence rate of hyperthyroidism was 17.5%, and the incidence of major complications was 3.5% (2/57).

**Conclusion:**

Microwave “Near-Total Ablation” is a safe and effective method for the treatment of refractory hyperthyroidism, with a high postoperative euthyroidism rate and a low incidence of hypothyroidism. This technique is expected to become an alternative to surgical thyroidectomy for hyperthyroidism.

## Introduction

Primary hyperthyroidism is a common endocrine disorder with a higher prevalence in females and a lower incidence in males, with an overall morbidity rate of approximately 3% (1). Antithyroid drugs (ATDs) are the first-line treatment, but they are associated with a long treatment course and a high recurrence rate after drug withdrawal. Hyperthyroidism is defined as refractory when patients cannot discontinue ATDs after more than 18 months of treatment or experience recurrence after drug withdrawal (2, 3). For these patients, radioactive iodine (¹³¹I) therapy or surgery is recommended. However, some patients refuse these treatments due to concerns about radiation exposure, high surgical trauma, and frequent postoperative complications (4, 5).

Thermal ablation techniques, including microwave ablation (MWA) have achieved remarkable therapeutic effects in the treatment of benign thyroid nodules and papillary thyroid microcarcinoma (6, 7). This technique has also been applied in the management of refractory hyperthyroidism, with reported remission rates of hyperthyroidism ranging from 40% to 70% after a single-session ablation in published studies (8, 9). As this approach is still in the preliminary stage of clinical application, there is no widely accepted standardized protocol, and various ablation strategies are being explored. Some studies have reported a strategy of ablating the majority of the glandular tissue while preserving only a small amount of tissue adjacent to the recurrent laryngeal nerve, trachea, and blood vessels, but this method is highly dependent on the operator’s technical expertise (10). Other studies have adopted an ablation strategy targeting 30% to 60% of the glandular tissue, which results in an excessive amount of residual thyroid tissue (11, 12). The Chinese expert consensus (13) recommends the “Near-Total Ablation” approach, which refers to nearly complete ablation of the thyroid gland while ensuring the safety of vital structures in the neck, including nerves, blood vessels, the trachea, and parathyroid glands. Although this method is theoretically feasible, its safety and efficacy still need to be verified by clinical studies, especially regarding the risk of high postoperative hypothyroidism caused by minimal residual tissue, which has not been reported in the existing literature. This study aims to investigate the efficacy and safety of ultrasound-guided microwave “Near-Total Ablation” for refractory hyperthyroidism, with a particular focus on the postoperative drug-free euthyroidism rate and the incidence of hypothyroidism.

## Materials and methods

### Study subjects

This study was approved by the Hospital Ethics Committee (approval No. NFEC-2022-214). The ablation procedure and potential complications were explained in detail to all patients preoperatively, and written informed consent was obtained from each participant.

A total of 60 patients with GD underwent ultrasound-guided microwave ablation between December 2020 and July 2024. The cohort included 5 males and 55 females, aged 18 to 66 years, with a mean age of 44.7 ± 12.4 years. Inclusion criteria: ① Patients with primary hyperthyroidism meeting the clinical diagnostic criteria (14); ② Patients who could not discontinue ATDs after more than 18 months of treatment or experienced recurrence after drug withdrawal; ③ Patients with concomitant thyroid nodules (long diameter ≥ 1.0 cm) underwent fine-needle aspiration biopsy with benign pathological results (Bethesda category II); ④ Patients refused surgical treatment or ¹³¹I therapy and voluntarily received microwave ablation. Exclusion criteria: ① Patients with hyperthyroidism and nodules diagnosed as atypia of undetermined significance, suspicious for malignancy, or malignant by fine-needle aspiration biopsy; ② Patients with goiter extending to the retrosternal space or accompanied by obvious compressive symptoms; ③ Patients with severe Graves’ orbitopathy; ④ Patients aged < 18 years or > 70 years; ⑤ Patients with coagulation dysfunction; ⑥ Patients with severe cardiopulmonary diseases.

### Equipment

A Nanjing Kangyou KY-2000 Microwave Therapeutic System with an emission frequency of 2450 MHz was used, with an ablation power set between 35 W and 40 W. A matched KY-2450B water-cooled microwave ablation needle (16G × 10 cm) with a 3 mm antenna length was connected to the microwave generator via a low-loss coaxial cable. A French Aixplorer Supersonic Imagine color Doppler ultrasound system with an SL15–4 high-frequency linear array probe was used for preoperative evaluation, intraoperative guidance, and postoperative follow-up examinations.

### Preoperative preparation

All patients achieved normal or near-normal thyroid function with ATD treatment preoperatively, with a heart rate < 90 beats per minute, and continued taking their medications on the day of the procedure. Preoperative examinations included complete blood count, Coagulation profile, electrocardiogram, chest X-ray, thyroid function, and thyroid antibody tests. Conventional ultrasound was performed to measure three mutually perpendicular maximum diameters (a, b, c) of each thyroid lobe, and the pre-ablation volume (V0) of each lobe was calculated using the formula V0 = π/6 × a × b × c (15). Color Doppler flow imaging was used to evaluate the color blood flow of the thyroid gland.

### Ablation procedure

Cervical local anesthesia combined with intravenous sedation was adopted for all procedures. 10 mg of diazepam was injected via the dorsal hand vein preoperatively for sedation. The patient was placed in the supine position with a pillow under the shoulder and the neck hyperextended to fully expose the anterior cervical region. After routine skin disinfection and sterile draping, an appropriate amount of 1% lidocaine was injected under ultrasound guidance into the space between the anterior and lateral capsules of the thyroid gland for local infiltration anesthesia. An appropriate amount of 5% glucose solution was then injected via the isthmus between the medial aspect of the gland and the tracheal wall to form a “hydrodissection” (width > 3 mm). Subsequently, a transverse isthmic puncture approach was used. First, 1% lidocaine was administered for infiltration anesthesia at the puncture site and along the puncture path. A thick needle was used for skin puncture, and the microwave needle was then accurately inserted under ultrasound guidance along the puncture path into the deepest and most distal posteromedial part of the gland(**Figure 1**). The continuous “moving-shot technique” was used for ablation (16). The majority of the glandular tissue of the bilateral thyroid lobes and isthmus was ablated, with only a small amount of glandular tissue preserved adjacent to the tracheal wall, recurrent laryngeal nerve, and carotid artery(**Figure 2**). Vascular injury was avoided during the puncture process. Ablation was terminated when the vaporization zone completely covered the predetermined ablation range. Color Doppler ultrasound was used to check for blood flow signals in the ablation zone; if present, supplementary ablation was performed immediately until the blood flow signals disappeared. Concomitant thyroid nodules were ablated together with the surrounding glandular tissue. After completing ablation of one thyroid lobe, ablation of the contralateral lobe was continued if the patient had no discomfort and normal phonation. If the patient developed hoarseness or other severe complications, the ablation was terminated, and the contralateral lobe was ablated after the symptoms resolved. Local compression was applied for 1 hour after the completion of ablation. The patient was then transferred to the ward for observation, and the ablation duration, total energy delivery, and complications were recorded.

**Figure 1:**
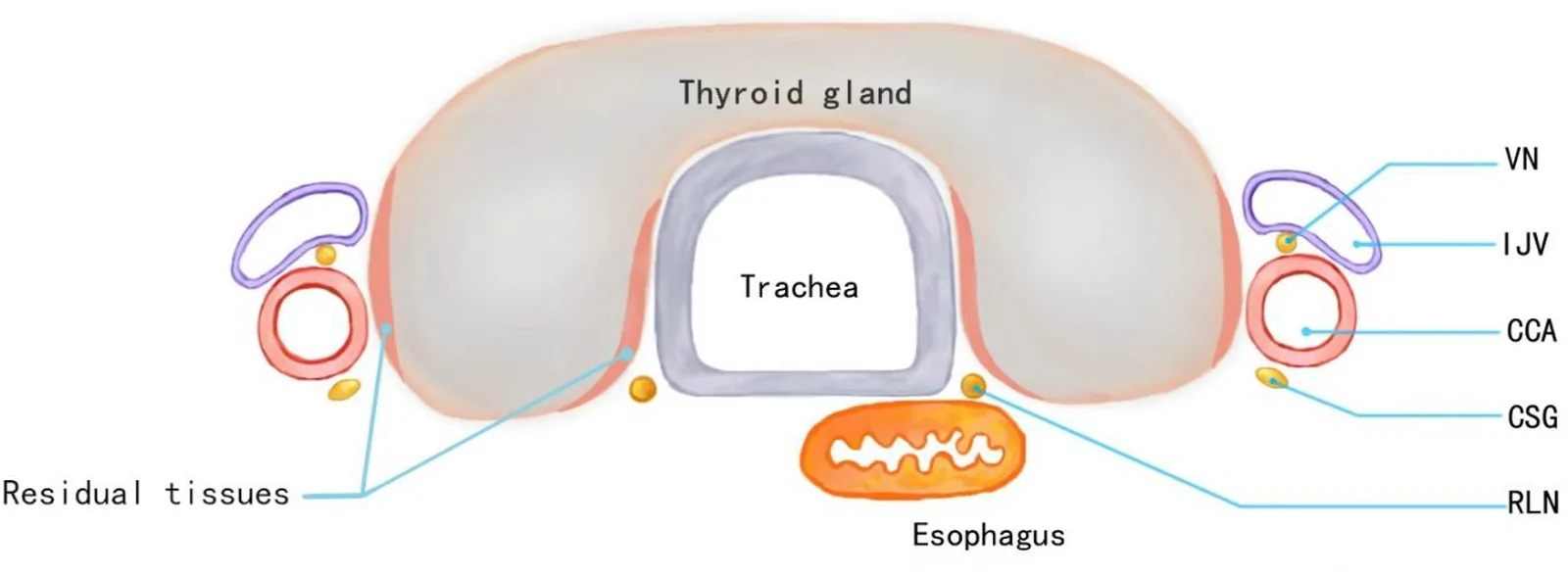
Schematic of thyroid gland and surrounding tissues. VN, Vagus nerve; IJV, internal jugular vein; CCA, common carotid artery; CSG, Cervical sympathetic ganglion; RLN, recurrent laryngeal nerve.

**Figure 2:**
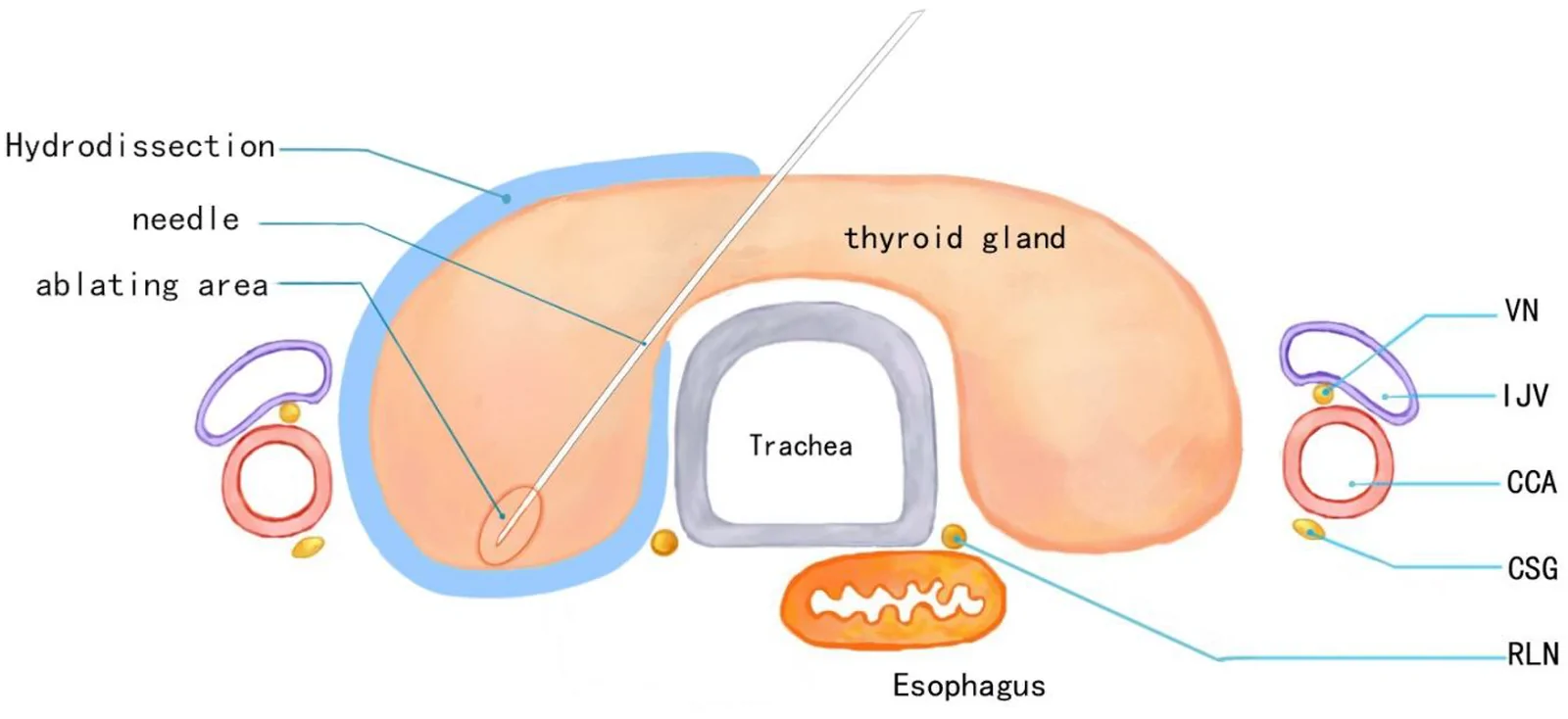
Schematic of Ultrasound-Guided Microwave “Near-Total Ablation” VN, Vagus nerve; IJV, internal jugular vein; CCA, common carotid artery; CSG, Cervical sympathetic ganglion; RLN, recurrent laryngeal nerve.

### Postoperative follow-up

ATD treatment was discontinued immediately after completion of ablation of the bilateral thyroid lobes and isthmus. Propranolol was continued for patients with palpitations and discontinued after the symptoms resolved. Follow-up visits were scheduled at 1, 3, and 6 months postoperatively, and then every 6 months thereafter, with additional visits if clinically necessary. Follow-up assessments included thyroid function tests (free triiodothyronine [FT3], free thyroxine [FT4], thyroid-stimulating hormone [TSH], and thyrotropin receptor antibody [TRAb]), clinical symptoms, and changes in thyroid volume (17). Levothyroxine (LT4) replacement therapy was initiated for patients who developed hypothyroidism during follow-up to restore normal thyroid function. For patients with unrelieved hyperthyroidism, recurrent hyperthyroidism after initial remission, or persistent subclinical hyperthyroidism (TSH < 0.10 mIU/L), supplementary microwave ablation was performed using the same protocol described above.

### Statistical analysis

Statistical analysis was performed using SPSS 25.0 software. Measurement data conforming to a normal distribution were expressed as mean ± standard deviation, and paired *t*-test was used for intergroup comparison. Measurement data not conforming to a normal distribution were expressed as median and interquartile range, and the Mann-Whitney *U* test was used for intergroup comparison. Count data were expressed as frequency and percentage, and *Chi-square* test or *Fisher’s* exact test was used for intergroup comparison. Univariate and multivariate Logistic regression analyses were performed to identify factors influencing therapeutic efficacy. A *P*-value < 0.05 was considered statistically significant.

## Results

Of the 60 enrolled patients, 3 were excluded from the final analysis: 1 patient underwent unilateral ablation and failed to undergo the scheduled contralateral ablation due to loss to follow-up after the initial procedure, and the other 2 patients were lost to postoperative follow-up. The remaining 57 patients completed ablation of the bilateral thyroid lobes and isthmus, including 5 males (8.8%) and 52 females (91.2%). The baseline characteristics of the patients before ablation are shown in **Table 1**. The duration of hyperthyroidism before the procedure ranged from 2 to 23 years, with a mean of 5.3 ± 4.1 years. Thyroid enlargement was grade Iin 20 patients (35.1%), grade IIin 29 patients (50.9%), and grade III in 8 patients (14.0%). Thirteen patients (22.8%) presented with mild to moderate exophthalmos, all with a Clinical Activity Score (CAS) of 0. All patients underwent “Near-Total Ablation” (**Figure 3**), with the ablation range covering the bilateral thyroid lobes and isthmus; concomitant nodules were ablated with the surrounding tissue. ATDs were discontinued postoperatively in all patients. Patients with preoperative palpitations continued propranolol treatment after the procedure, which was discontinued once the heart rate normalized.

**Table 1:** Baseline characteristics of patients undergoing microwave ablation for refractory hyperthyroidism.

Charecteristics	Mean ± SD/median (IQR), n (%)
Age at treatment (years)	35.5 ± 12.2
Sex, n (%)	
Male	5(8.8%)
Female	52(91.2%)
Disease duration (years)	4.0 (2.0, 8.0)
heart rate before ablation (beats/min)	79.7 ± 11.7
Daily maintenance dose of carbimazole before MWA	
<10 mg	18 (31.6%)
10∼20 mg	30 (52.6%)
>20 mg	9 (15.8%)
Unilateral or bilateral thyroid nodules(FNA,BethesdaII)	36(63.2%)
Mild-to-moderate exophthalmos	13(22.8%)
Thyroid enlargement grade (by WHO classification)	
Grade I°	20(35.1%)
Grade II°	29(50.9%)
Grade III°	8(14.0%)
Serum preablation Free T3 (pg/ml)	4.62 ± 3.62
Serum preablation Free T4 (ng/dL)	1.15 ± 0.53
Serum preablation TSH (mIU/L)	0.01 (0.00, 0.15)
Serum preablation TRAb (IU/L)	9.51 (3.50, 24.33)

IQR, Interquartile Range; FNA, Fine-Needle Aspiration; MWA, Microwave Ablation; WHO, World Health Organization.

**Figure 3:**
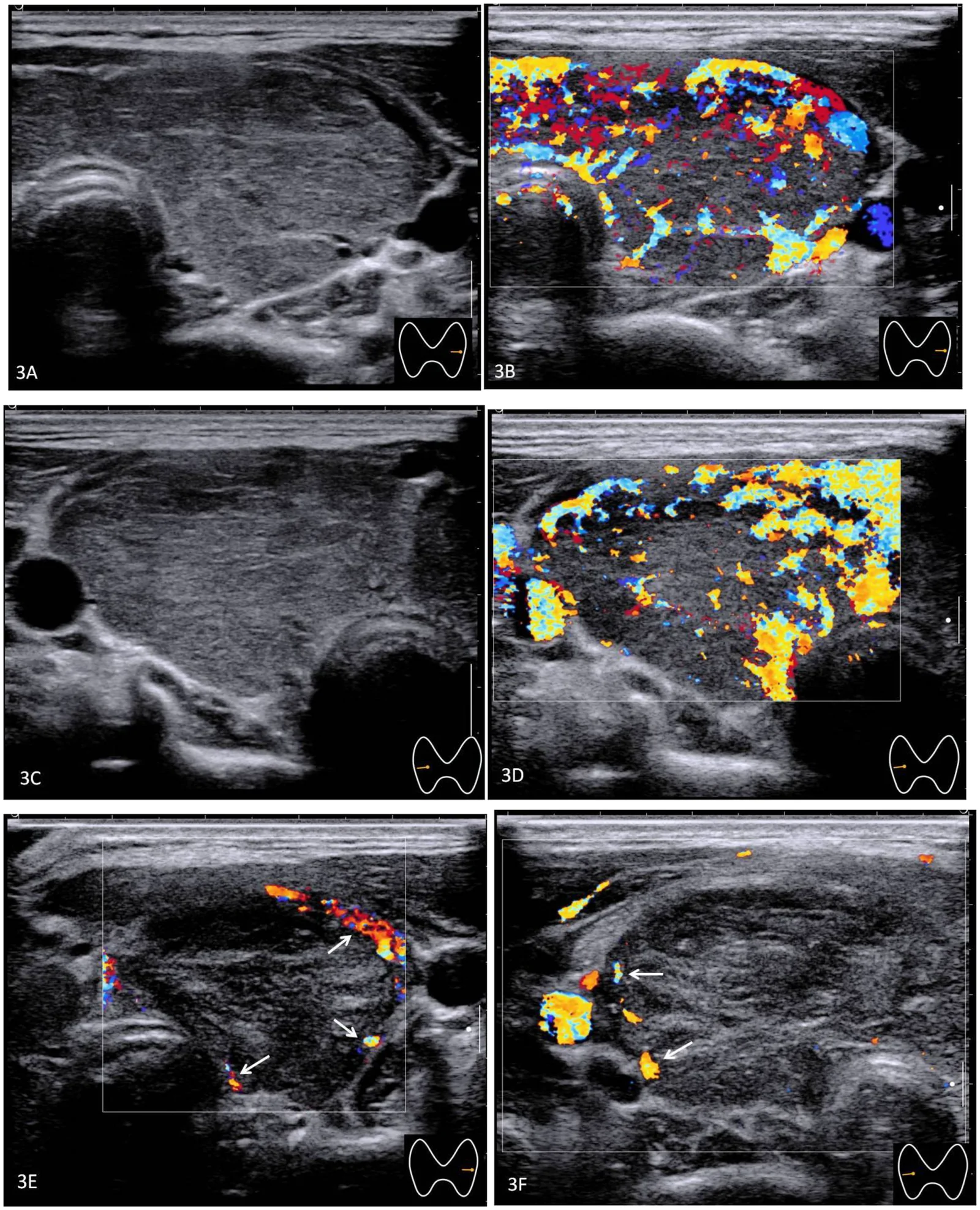
Ultrasonographic findings of diffuse thyroid lesions before and after microwave ablation. **(A)** Conventional 2D ultrasound shows diffuse enlargement and heterogeneous echogenicity of the left lobe and isthmus of the thyroid. **(B)** Color Doppler flow imaging reveals extremely abundant blood flow signals in the left lobe and isthmus of the thyroid. **(C)** Conventional 2D ultrasound shows diffuse enlargement and heterogeneous echogenicity of the right lobe of the thyroid. **(D)** Color Doppler flow imaging reveals extremely abundant blood flow signals in the right lobe of the thyroid. **(E)** Postoperative ultrasound after microwave ablation of the left thyroid lobe. Most parenchymal tissue was ablated with no blood flow signal. A small amount of residual tissue at the margin shows blood flow on CDFI (arrows). **(F)** Postoperative ultrasound after microwave ablation of the right thyroid lobe and isthmus. Most parenchymal tissue was ablated with no blood flow signal. A small amount of residual tissue at the margin shows blood flow on CDFI (arrows).

The follow-up duration ranged from 6 to 36 months, with a mean of 10.6 ± 4.6 months. During follow-up, 35 patients achieved hyperthyroidism remission (normal FT3 and FT4 levels), with a remission rate of 61.4%, and were assigned to the remission group. The remaining 22 patients had unrelieved hyperthyroidism (elevated FT3 and FT4 with decreased TSH), with a non-remission rate of 38.6%, and were assigned to the non-remission group. Comparison of baseline data between the two groups is shown in **Table 2**. There were statistically significant differences in thyroid volume, goiter grade, total ablation energy, ablation duration, preoperative ATD dosage, and TSH level between the two groups (*P* < 0.05). The non-remission group had significantly higher values for all the above parameters except TSH, which was significantly lower than that in the remission group. Univariate Logistic regression analysis demonstrated that thyroid volume, goiter grade, total ablation energy, ablation duration, preoperative ATD dosage, and preoperative TSH level were significantly correlated with therapeutic efficacy. Notably, total ablation energy and ablation duration are intraoperative characteristic parameters determined by baseline thyroid volume and goiter grade. Further multivariate Logistic regression analysis confirmed that only thyroid volume, goiter grade, preoperative ATD dosage, and preoperative TSH level were independent influencing factors for therapeutic efficacy (*P* < 0.05), while total ablation energy and ablation duration were no longer independent predictors after adjusting for baseline thyroid morphological confounders. Gender, age, disease duration, preoperative heart rate, FT3, FT4, and TRAb levels had no significant impact on therapeutic efficacy (*P* > 0.05) (**Table 3**).

**Table 2:** Comparison of preoperative parameters between the remission group and non-remission group.

Charecteristics	Remission group (n=35)	Non-remission group (n=22)	*P* value
Sex, Male/Female	3/32	2/20	1.000
Age (years)	38.6 ± 10.2	39.2 ± 9.8	0.895
Disease duration (months)	3.6 ± 1.8	4.2 ± 2.6	0.111
Heart rate (beats/min)	92.5 ± 12.8	98.6 ± 15.2	0.089
Daily MMI maintenance dose (mg)	12.5 ± 7.8	21.8 ± 9.2	0.001*
Thyroid enlargement grade	1.32 ± 1.01	1.95 ± 0.90	0.044*
Serum preablation Free T3 (pg/ml)	4.06 ± 2.06	5.51 ± 4.93	0.427
Serum preablation Free T4 (ng/dl)	1.09 ± 0.44	1.24 ± 0.63	0.412
Serum preablation TSH (mIU/L)	0.006 (0.002, 0.438)	0.003 (0.001, 0.005)	0.017*
Serum preablation TRAb (IU/L)	6.67 (2.290, 21.525)	13.30 (7.643, 22.575)	0.115
Total thyroid volume (ml)	28.42 ± 12.58	45.75 ± 18.31	0.001*
Total ablation energy (J)	68520 ± 24150	118640 ± 32580	0.001*
Total ablation duration (min)	45.13 ± 14.53	66.11 ± 19.42	0.001*

*P < 0.05; MMI, Methimazole.

**Table 3:** Results of univariate and multivariate logistic regression analysis of factors affecting ablation efficacy.

Variables	Univariate logistic regression OR (95% CI)	Univariate *P* value	Multivariate logistic regression OR (95% CI)	Multivariate *P* value
Age (years)	1.00(0.95~1.05)	0.896	–	–
Sex (Female/Male)	0.92(0.14~6.01)	1.000	–	–
Total Thyroid volume (ml)	1.045(1.012-1.079)	0.008**	1.05(1.01~1.08)	0.008*
Total Ablation duration (min)	1.00(1.00~1.00)	0.003**	–	–
Total Ablation energy (J)	1.00(1.00~1.00)	0.001**	1.00(1.00~1.00)	0.068
Disease duration (months)	1.06(0.98~1.15)	0.112	–	–
MMI dose (mg)	1.092(1.018-1.171)	0.015**	1.09(1.02~1.17)	0.015*
Thyroid enlargement grade	3.124(1.285-7.596)	0.012**	3.12(1.29~7.60)	0.012*
Heart rate (beats/min)	1.028(0.998-1.059)	0.065	1.03(0.99~1.06)	0.105
FT3 (pg/ml)	1.05(0.94~1.17)	0.427	–	–
FT4 (ng/dL)	1.45(0.59~3.55)	0.412	–	–
TSH (mIU/L)	0.286(0.105-0.782)	0.014*	0.29(0.11~0.78)	0.014*
TRAb (IU/L)	1.015(0.998-1.032)	0.072	1.01(0.99~1.03)	0.089

*P < 0.05, **P < 0.01. OR, Odds Ratio; CI, Confidence Interval.

TSH distribution in the 35 patients of the remission group was as follows: elevated in 5 patients, normal in 17 patients, and decreased in 13 patients. During follow-up, patients with elevated TSH received LT4 replacement therapy, and those with normal thyroid function were followed up regularly. Patients with persistent TSH < 0.1 mIU/L and those with recurrent hyperthyroidism after initial remission underwent supplementary microwave ablation. During the 6- to 36-month follow-up period, 4 patients with persistent TSH < 0.1 mIU/L and 8 patients with recurrent hyperthyroidism achieved normal thyroid function after a single session of supplementary microwave ablation.

Among the 22 patients in the non-remission group, 18 achieved hyperthyroidism remission after 1 session of supplementary ablation, 3 after 2 sessions, and 1 after 3 sessions of supplementary microwave ablation during follow-up. Corresponding management was provided according to TSH changes during follow-up, using the same protocol as described above. Two patients experienced hyperthyroidism recurrence after initial remission: 1 achieved normal thyroid function after 1 session of supplementary ablation, and the other chose to continue ATD treatment.

A total of 10 patients experienced recurrent hyperthyroidism after initial remission during follow-up, with a recurrence rate of 17.5%. The time to recurrence ranged from 3 months to 36 months postoperatively, with a median of 11 months. At the last follow-up visit, hyperthyroidism remission was achieved in 56 of the 57 patients, with a final remission rate of 98.2%; 1 patient with recurrent hyperthyroidism switched to ATD treatment, with a non-remission rate of 1.8%. Thirty-nine patients (68.4%) had normal thyroid function without medication dependence, and 17 patients (29.8%) had hypothyroidism requiring LT4 replacement therapy. There was no significant change in exophthalmos during follow-up, with the CAS remaining 0 in all affected patients.

Major complications occurred in 2 patients in this cohort, with an incidence of 3.5%, both presenting with hoarseness. Two patients developed transient hoarseness after bilateral ablation. Hoarseness manifested in the days following ablation, and vocal function returned to normal spontaneously within 1–3 months without further invasive treatment. Minor complications occurred in 2 patients, with an incidence of 3.5%, both presenting with subcapsular hemorrhage. The bleeding was stopped by cervical compression, and the symptoms resolved within 1 week postoperatively. No complications such as tracheal injury, esophageal injury, or skin burn occurred. Other transient symptoms, including mild intraoperative pain and radiating pain in the retroauricular and shoulder-back regions, were tolerable, did not affect the ablation procedure, and resolved immediately after the completion of ablation.

## Discussion

Microwave ablation for refractory hyperthyroidism using the “Near-Total Ablation” approach is defined as the inactivation of the majority of the glandular tissue of the bilateral thyroid lobes and isthmus, with only a small amount of glandular tissue preserved adjacent to the recurrent laryngeal nerve, trachea, and carotid artery to avoid injury to these vital structures (13). Inactivation of the majority of the thyroid parenchyma reduces the synthesis and secretion of thyroid hormones, thereby achieving remission of hyperthyroidism. The results of this study demonstrate that “Near-Total Ablation” is effective for the treatment of refractory hyperthyroidism, with a hyperthyroidism remission rate of 61.4% after a single session ablation, which is consistent with the remission rates of 40% to 70% reported in previous literature (8, 9).

Due to factors such as the operator’s technical expertise and individual patient variability, even with the same ablation protocol, hyperthyroidism cannot be relieved in all patients, and postoperative non-remission of hyperthyroidism is a common occurrence. The non-remission rate after a single ablation in this study was 38.6%, which is comparable to the non-remission rates of 20% to 40% reported in the literature (8, 9, 12). For these patients, supplementary microwave ablation can be performed, or ATD, ¹³¹I therapy can be selected according to the patient’s preference. In this cohort, only 1 patient with recurrent hyperthyroidism after ablation chose to continue ATD treatment, while all other patients with unrelieved or recurrent hyperthyroidism underwent supplementary microwave ablation. After 1 to 3 sessions of supplementary ablation, the hyperthyroidism remission rate increased from 61.4% to 98.2%. Compared with surgery, microwave ablation has the advantages of minimal invasiveness and repeatability, giving it certain advantages in the treatment of hyperthyroidism.

Other factors can affect the therapeutic effect of hyperthyroidism ablation, among which gland size is the dominant factor. Fung et al. (17) reported that the remission rate after a single session ablation reached 100% in patients with a total thyroid volume < 20 mL, while the remission rate was only 35% in those with a total volume ≥ 20 mL. Lang et al. (18) reported that the smaller the thyroid gland, the lower the risk of postoperative recurrence, and vice versa. The results of this study also verified that the postoperative remission rate is correlated with gland size: the thyroid volume in the non-remission group was significantly larger than that in the remission group, and there were statistically significant differences in goiter grade, volume, ablation duration, and total ablation energy (all parameters related to gland size) between the two groups (*P* < 0.05). The underlying reason may be that a larger gland requires more tissue to be ablated, resulting in a longer ablation duration and more total energy delivery. In addition, due to the interference of hyperechoic gas and the heat sink effect during ablation, the amount of residual glandular tissue adjacent to blood vessels and the thyroid capsule increases, and the actual residual tissue volume is higher than the preoperative planned volume, which impairs the therapeutic effect of ablation.

Remission of hyperthyroidism after ablation does not equal euthyroidism, and may also present as hypothyroidism or subclinical hyperthyroidism. These thyroid function states are not stable, and corresponding management should be implemented according to changes in thyroid function during follow-up. Patients with hypothyroidism require LT4 replacement therapy. For subclinical hyperthyroidism, according to clinical guidelines (19), regular follow-up without treatment is recommended when TSH ≥ 0.10 mIU/L, while when TSH < 0.10 mIU/L, there is a risk of atrial fibrillation, reduced bone mineral density, and fractures, and supplementary ablation is required to restore TSH to ≥ 0.10 mIU/L or normal levels. After these interventions, at the last follow-up visit in this study, 68.4% of patients achieved complete euthyroidism without medication dependence, 29.8% of patients had hypothyroidism requiring LT4 replacement therapy, and the hyperthyroidism recurrence rate was 17.5%.

Ultrasound-guided thyroid “Near-Total Ablation” is different from surgical “near-total thyroidectomy” (20). The latter preserves < 1 g of residual glandular tissue, resulting in a 100% incidence of hypothyroidism requiring long-term LT4 replacement therapy. The results of this study show that the therapeutic effect of “Near-Total Ablation” is similar to that of surgical “subtotal thyroidectomy”. Sugino et al. (21) reported that after surgical subtotal thyroidectomy for hyperthyroidism, the euthyroidism rate was 17.4%, the hypothyroidism rate was 72.3%, and the recurrence rate was 10.3%. In comparison, “Near-Total Ablation” appears to have a superior euthyroidism rate to surgical subtotal thyroidectomy, with a similar recurrence rate.

Thyroid ablation has certain advantages compared with traditional treatment methods for hyperthyroidism. Compared with ATDs, microwave ablation has a shorter treatment course and faster onset of effect, with a 1-year hyperthyroidism remission rate > 95% and a recurrence rate of only 17.5%. In contrast, ATD treatment requires a long treatment cycle, with a recurrence rate of over 50% after drug withdrawal (22). Compared with ¹³¹I therapy, microwave ablation has a lower incidence of hypothyroidism, at only 29.8% in this study. The 1-year incidence of hypothyroidism after ¹³¹I therapy is approximately 70%, with an annual increase of 3% thereafter; in addition, there is a risk of radiation exposure and exacerbation of Graves’ orbitopathy (23). Compared with surgery, microwave ablation has the advantages of minimal invasiveness and no cervical scarring. The incidence of hypothyroidism after total or near-total thyroidectomy for hyperthyroidism is 100%, requiring lifelong LT4 replacement therapy (24). The remission rate after subtotal thyroidectomy for hyperthyroidism is approximately 70% (25), and due to the high risk of reoperation, most patients with unrelieved or recurrent hyperthyroidism after surgery choose ¹³¹I therapy or medical treatment.

The results of this study demonstrate that “Near-Total Ablation” has a favorable safety profile, with a major complication rate of only 3.5%, which is similar to the complication rate of thyroid nodule ablation (26, 27). The low complication rate may be attributed to multiple protective measures adopted during the procedure: in addition to hydrodisection, a small amount of glandular tissue was preserved adjacent to blood vessels and nerves to avoid thermal injury. The needle track ablation technique was used during puncture to achieve hemostasis and prevent vascular puncture by the needle tip (28).

## Limitations

First, the follow-up duration was relatively short for some patients in this study, and extended follow-up is beneficial for a more comprehensive evaluation of the long-term therapeutic efficacy. Second, this study did not assess changes in patients’ quality of life before and after ablation, which will be included in future studies. Third, this is a single-center retrospective study, and prospective multicenter studies are needed to further verify the efficacy and safety of this technique in the future.

## Conclusion

Microwave “Near-Total Ablation” is a safe and effective method for the treatment of refractory hyperthyroidism, with a high postoperative drug-free euthyroidism rate and a low incidence of hypothyroidism. This technique is expected to become an alternative to surgical thyroidectomy.

## Data Availability

The original contributions presented in the study are included in the article/supplementary material. Further inquiries can be directed to the corresponding author.
